# Supplementary material for: Characterization of Loss-of-Imprinting in Breast Cancer at the Cellular Level by Integrating Single-Cell Full-Length Transcriptome with Bulk RNA-Seq Data
Source: Biomolecules. 2024 Dec 14;14(12):1598. doi: 10.3390/biom14121598 (PMC11673884; doi:10.3390/biom14121598)
Supplement: Supplementary file 1 [file biomolecules-14-01598-s001.zip › Supplementary figure.pdf]

Characterization of Loss-of-Imprinting in Breast Cancer at the Cellular Level by Integrating Single-Cell Full-Length Transcriptome with Bulk RNA-Seq Data

Muhammad Talal Amin [1,2,3], Louis Coussement [1,2], Tim De Meyer [1,2,4*]

1. Department of Data Analysis and Mathematical Modelling, Faculty of Bioscience Engineering, Ghent University, Ghent, Belgium

2. Cancer Research Institute Ghent (CRIG), Ghent, Belgium

3. Department of Bioscience and Technology, Khwaja Fareed University of Engineering and Information Technology, Rahim Yar Khan, Pakistan

4. Bioinformatics Institute Ghent N2N, Ghent University, Ghent, Belgium

* tim.demeyer@ugent.be

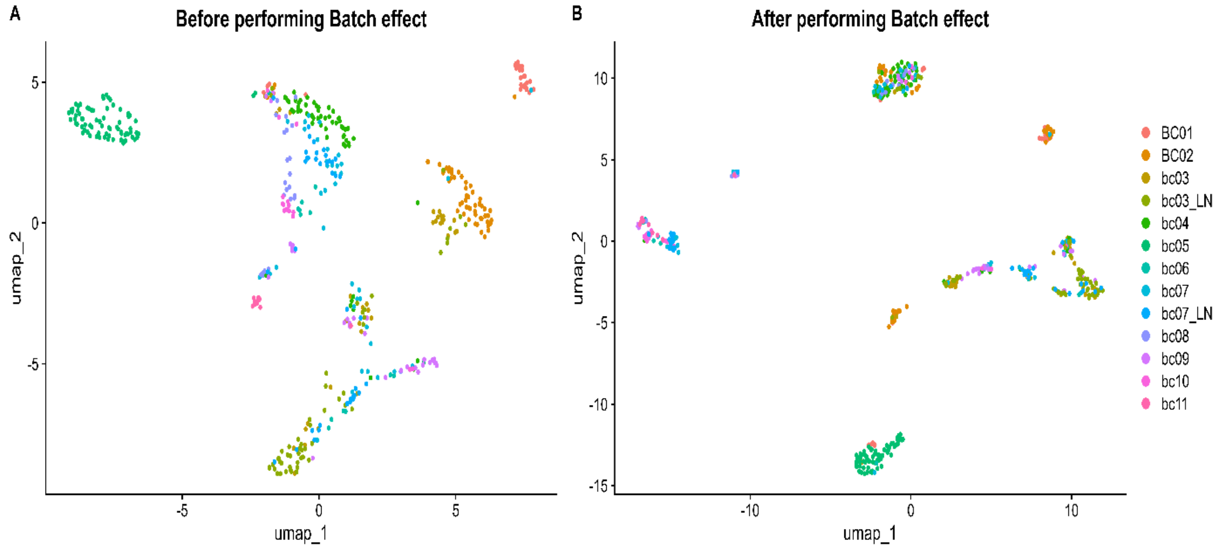

Fig S1 *Comparative analysis of batch effects in sc-RNA sequencing data.* The left plot **(A)** illustrates the distribution of cells before batch correction, with distinct clusters indicating technical variability. The right plot **(B)** shows the same data after batch correction (Harmony), where the cells are clustered together on biological variability. Each color represents a different batch (patient), as indicated by the legend. The x-axes and y-axes represent the UMAP dimensions, which help visualize the data in a reduced dimensional space. This comparison highlights the importance of batch correction in ensuring accurate and reliable single-cell RNA sequencing analysis.

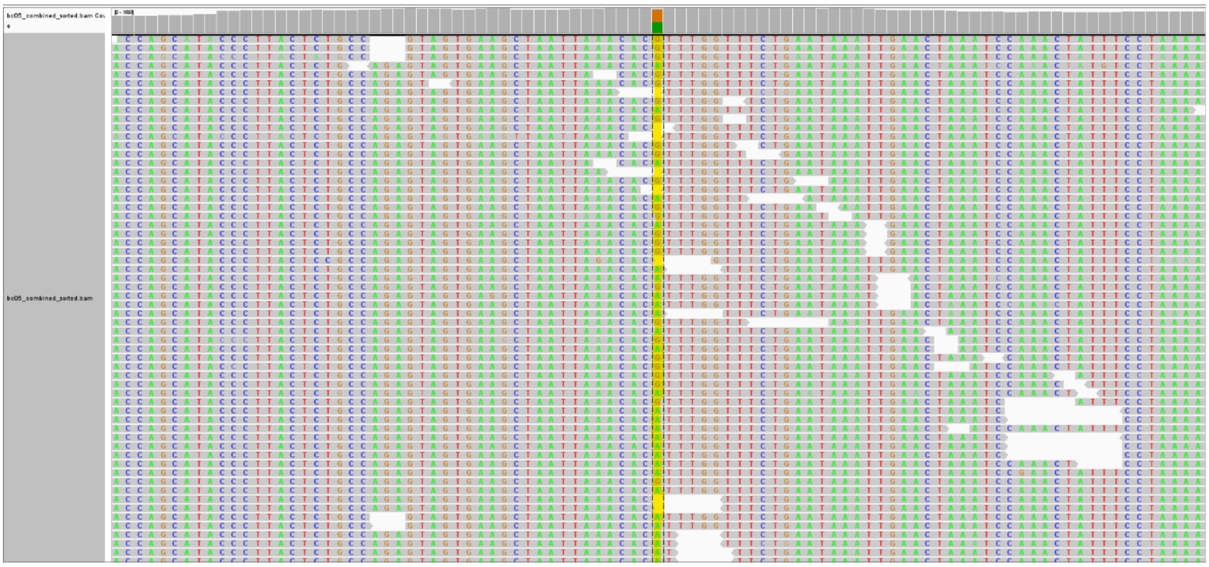

Fig S2 *Biallelic Expression of SNP rs10863 in MEST for Patient BC05*. The snapshot as observed in IV Genome Browser highlights representative subset of scRNA-seq reads aligned to LOI featuring MEST SNP (rs10863) merged over all BC05 cancer cells. The snapshot depicts allele-specific representative read counts (94 bp window), which confirm the presence of both alleles in the transcriptome, indicative of loss of imprinting (LOI) at this locus.
